# Urothelial Carcinoma With Bone Metastasis Mimicking Sciatica: A Common Neoplasm With an Uncommon Presentation

**DOI:** 10.7759/cureus.55259

**Published:** 2024-02-29

**Authors:** Ravleen Kaur, Lavleen Kaur, Aimen Iqbal, Nirali Patel

**Affiliations:** 1 Internal Medicine, The Wright Center for Graduate Medical Education, Scranton, USA

**Keywords:** bladder tumor, chronic lower back pain, uncommon presentation, common neoplasm, sciatica, bone metastasis, urothelial malignancy

## Abstract

Bone metastasis in urothelial cancer is underreported and not well-researched. A case of urothelial carcinoma (UC) with bone metastasis presenting as musculoskeletal pain is reported. The patient presented with persistent lower back pain associated with right lower extremity pain, numbness, and tingling. Initially, a diagnosis of sciatica was suspected, but the patient did not respond to treatment. An MRI spine was done, which revealed a bright signal mass in the vertebral body suspicious for a metastatic lesion, left hydroureteronephrosis, and a nonspecific cystic focus in the right iliacus muscle. Subsequent imaging revealed an irregular soft tissue mass at the left posterolateral bladder base, resulting in apparent obstruction of the left ureter, highly suggestive of neoplasm, along with numerous lytic bone lesions in the pelvic girdle with associated soft tissue masses, consistent with metastatic disease. The patient underwent an interventional radiology biopsy of the right iliac soft tissue mass to evaluate the lytic bony lesions, which revealed metastatic carcinoma, consistent with UC. A prompt referral was made for urology and oncology consultations. The patient underwent left percutaneous nephrostomy placement for obstruction, but he was not a candidate for any systemic therapy because of his poor performance status, and hospice was recommended as his metastatic disease was not curable and the goal of any kind of treatment was palliative. The optimal treatment for UC with bone metastasis remains divergent, and the management options should be determined as part of a shared decision-making process. This case highlights the importance of having a high suspicion of neoplastic pathology in patients presenting with musculoskeletal pain, like back pain, and not responding to treatment. This should alert the physicians to the potential for serious disease processes.

## Introduction

Bladder cancer has high mortality and morbidity and is the 12th most common cancer worldwide and the 6th most common in the United States. Urothelial carcinoma (UC) is the most common type of bladder cancer. About 25% of patients present with metastatic disease with only a 4.6% five-year survival rate. The incidence of bone metastasis in bladder cancer is between 1.39 to 5.5% as synchronous metastatic disease and 30 to 40% as metasynchronous metastatic disease [1]. UC metastasis has a male predominance (60%) with a median age of 66.5 years in the patients [2]. Typical bone metastasis is osteolytic (70%), but UC can also cause osteoblastic lesions (10%), although rare [3]. The usual initial symptom is hematuria and flank pain in one-third of patients [4]. Metastatic bone disease can present with diverse clinical signs and symptoms, including mechanical, non-mechanical, and referred pain or neurological dysfunction secondary to cord or nerve root compression [5]. Non-metastatic UC management is based on local excision, and depending on the relapse risk, it is often associated with adjuvant local chemotherapy or Bacille Calmette-Guerin instillation. Metastatic UC is treated with chemotherapeutic agents, but the prognosis remains poor. The approval of immunotherapy and targeted therapies has demonstrated better outcomes compared to chemotherapy as a second or subsequent line of treatment [6]. However, the median overall survival of patients with metastatic UC varies from < 10 to > 15 months, based on the treatment [7]. We present a case of urothelial bladder carcinoma with synchronous bone metastasis in an elderly male presenting low back pain.

## Case presentation

**Figure 1:**
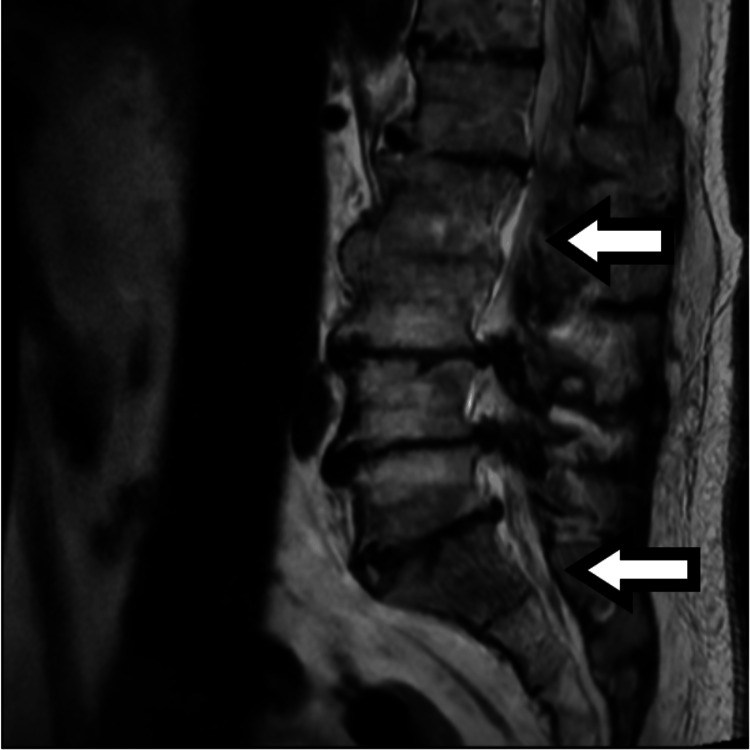
MRI of lumbosacral spine Sagittal view showing acute insufficiency fractures involving S1, S2, and left sacral ala, T1 low and T2 bright signal mass in L3 vertebral body suspicious for a metastatic lesion. MRI: magnetic resonance imaging

**Figure 2:**
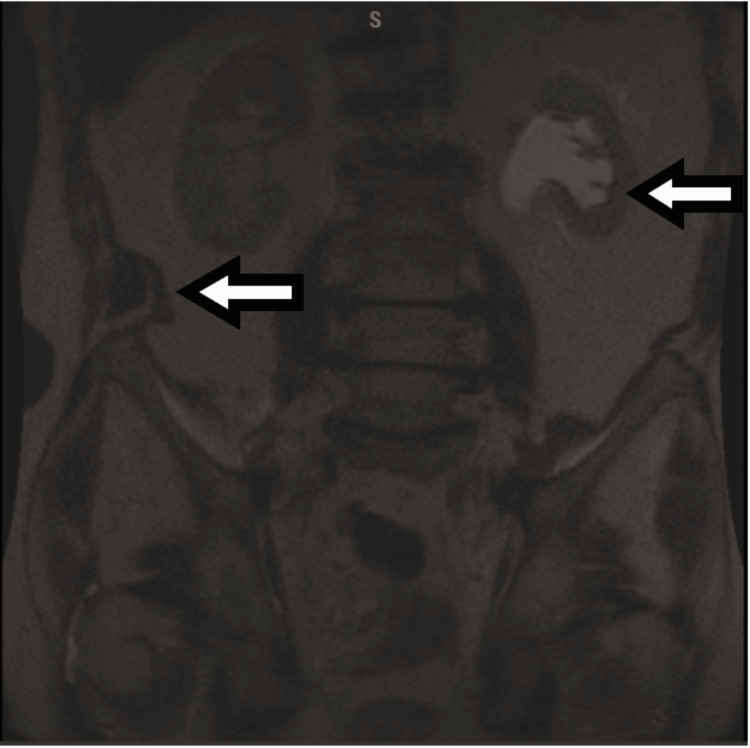
MRI of the spine Coronal view showing moderate to severe left hydroureteronephrosis, a nonspecific 11 mm cystic focus in the right iliacus muscle. MRI: magnetic resonance imaging

An 86-year-old male with a past medical history significant for hypertension, kidney stones, and hypothyroidism presented to the ED for evaluation of the sudden onset of worsening of his chronic lower back and right lower extremity pain and numbness ongoing for the last nine to 10 months. The back pain was described as moderate in intensity, radiating to the right lower extremity, progressively getting worse, and associated with lower extremity tingling, numbness, and ambulatory dysfunction. He was initially suspected to have sciatica but was not responding to treatment. The patient denied a history of falls, trauma, saddle anesthesia, urinary or bowel incontinence, urinary frequency, urgency, or strain, and hematuria. In the ED, the patient was found to be hemodynamically stable and saturating well on room air. Lab work was significant for leukocytosis with a WBC count of 20.57, hemoglobin of 10.5, acute kidney injury with blood urea nitrogen of 44, creatinine of 1.7 (baseline 1.1), and lactate of 3.7. Urinalysis was significant for pyuria and microscopic hematuria. The chest X-ray was negative for acute cardiopulmonary disease. MRI spine findings were consistent with acute insufficiency fractures involving S1, S2, and left sacral ala, T1 low, and T2 bright signal mass in the L3 vertebral body suspicious for a metastatic lesion. Moderate to severe left hydroureteronephrosis, a nonspecific cystic focus in the right iliacus muscle, multilevel degenerative disc disease causing spinal canal and neural foraminal stenosis (Figures 1-2).

**Figure 3:**
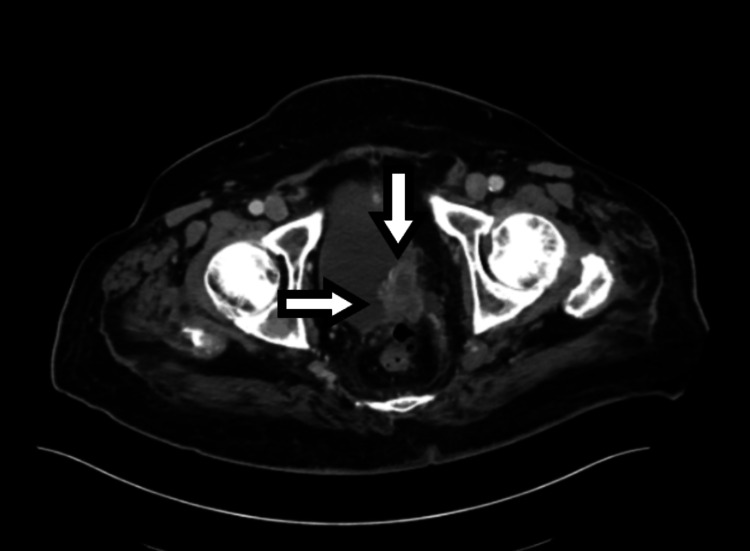
A CT scan of the abdomen and pelvis demonstrated an irregular soft tissue mass at the left posterolateral bladder base, involving the ureterovesical junction, highly suggestive of neoplasm in transverse view CT: computed tomography

This was followed by a CT scan of the abdomen and pelvis, which revealed an irregular soft tissue mass at the left posterolateral bladder base, involving the ureterovesical junction and resulting in apparent obstruction of the left ureter, highly suggestive of neoplasm, along with numerous lytic bone lesions in the pelvic girdle with associated soft tissue masses, consistent with metastatic disease (Figure 3). The CT head showed no acute intracranial abnormality with no signs of metastasis.

**Figure 4:**
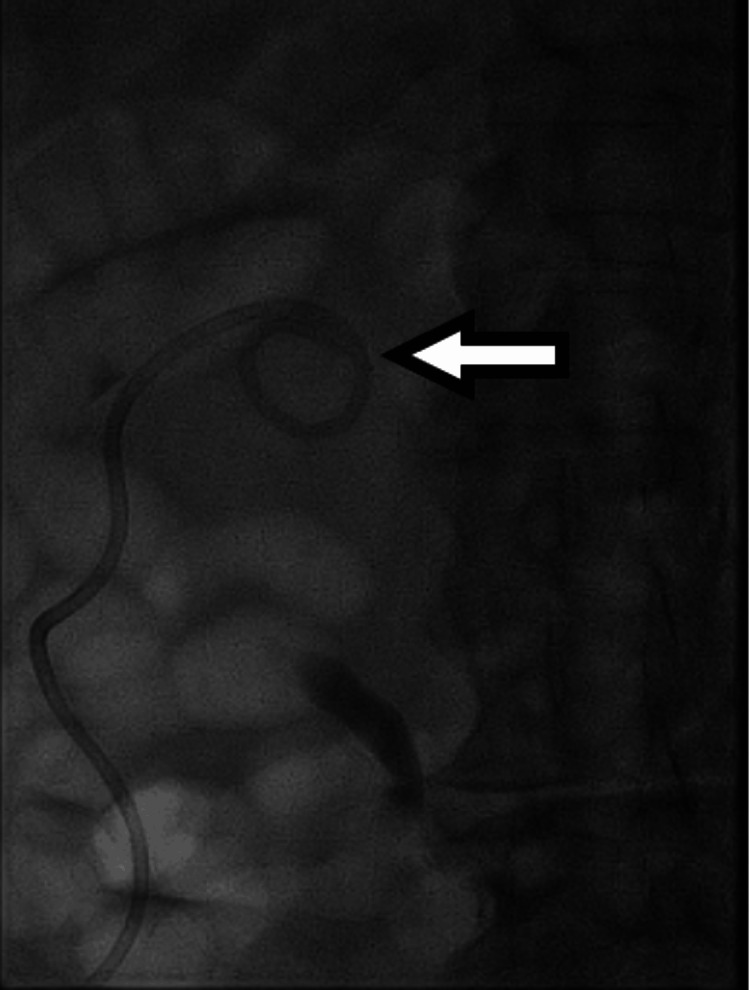
Ultrasound imaging demonstrated successful image-guided percutaneous placement of a left nephrostomy tube for moderate left hydronephrosis

**Figure 5:**
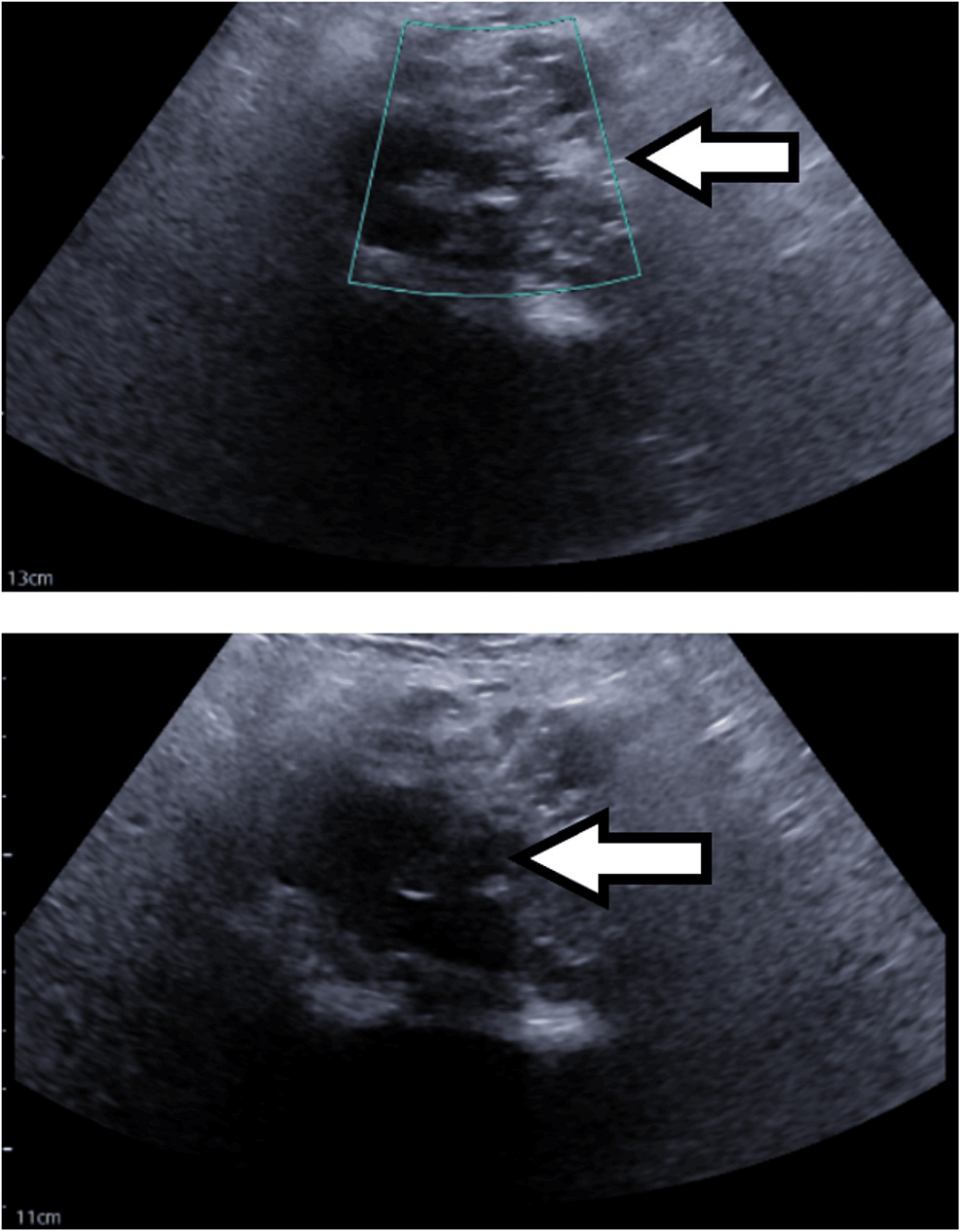
Ultrasound-guided percutaneous right iliac soft tissue mass biopsy

Orthopedics was consulted, who recommended IV decadron 20 mg followed by 10 mg IV decadron eight hours later, x 2 doses eight hours apart, with no surgical intervention. Urology was on board, and the patient underwent left percutaneous nephrostomy placement for obstruction (Figure 4) and was started on antibiotics for urinary tract infection. The patient underwent an interventional radiology biopsy of the right iliac soft tissue mass to evaluate the lytic bony lesions (Figure 5). The biopsy results revealed metastatic carcinoma, consistent with UC. The tumor cells were positive for CK7, CK20, P40, Uroplakin, and Gata-3, and showed focal positivity for CK5. The cytomorphology and immunoprofile were consistent with UC.

Hematology/oncology was consulted for UC with bone metastasis. The patient was advised to follow up as an outpatient for the initiation of therapy, either dual therapy or single therapy, with pembrolizumab given the patient's poor performance. Because of the patient's age, underlying comorbidities, and poor performance status, hospice was recommended as his metastatic disease was not curable and the goal of any kind of treatment was palliative. He was not a candidate for any systemic therapy because of his poor performance status. The patient and family agreed.

## Discussion

The most common neoplasm originating from the genitourinary system is UC. It is divided into the upper tract (involving the renal pelvis and ureter) UC and the lower tract (involving the bladder and urethra) UC. It can also be classified into low-grade (grade 1) and high-grade (grades 2 and 3) lesions depending upon the oncological behaviors, according to the WHO [2]. The presence of bone metastasis with or without pulmonary or liver involvement is defined as visceral metastasis from UC, which is recognized as a negative prognostic factor in a model developed at the Memorial Sloan Kettering Cancer Center and Karnofsky performance status in patients receiving cisplatin-based chemotherapy [1]. The principal metastatic sites are lymph nodes, followed by the liver, lung, and bone. The most common sites of bone metastasis are the pelvis (68%), spine (cervical 12%, thoracic 38%, and lumbar 34%), ribs (24%), and femur (22%) [1]. UC is associated with about 10.1% of the metastasis rate, has a poor prognosis, and is uniformly fatal. The median overall survival varies from 5.4 to 15 months, and 10.8 months for bone metastasis as the only metastatic site in urothelial metastasis [2]. Spinal metastasis is relatively rare, accounting for about 58.3% of all UC metastases. UC spinal metastasis is usually seen on radiological images like CT and bone scintigraphy as pathological fractures and soft tissue masses with dura matter compression [3].

Most commonly, bladder carcinoma presents with hematuria, signs of bladder irritation like urinary frequency or urgency, signs of obstruction like an intermittent stream, incomplete voiding or straining, and flank pain in advanced disease because of urethral obstruction, with painless hematuria being the most common symptom in patients with UC. It rarely presents as physical pain in the lower back or buttock or at a distant bone metastasis site at initial presentation without the usually reported symptoms [5].

Platinum-based chemotherapy is the first-line therapy, while immunotherapy is the second-line setting after the failure of first-line chemotherapy and as maintenance therapy in patients responding to first-line chemotherapy and first-line cisplatin-ineligible PD-L1-positive patients [6]. The overall survival outcome probability is precisely dependent on baseline patient and disease-related factors [7]. The presence of bone metastasis is an independent poor prognostic factor that negatively impacts overall survival along with others, which include WBC count, low hemoglobin levels, high levels of lactate dehydrogenase, C-reactive protein, low albumin level, patient performance status, and response to platinum-based chemotherapy [8]. A multidisciplinary approach is important in such cases involving different specialties in the areas of oncology, orthopedics, urology, psychology, anesthesiology, and radiotherapy. The patients should understand the long-term prognosis and treatment options available [9]. These patients demand special attention as bone metastasis has a less favorable prognosis as compared to other metastatic sites [10] and also due to the high rate of skeletal-related events like severe spinal instability, intractable pain, and poor life quality, which is a big factor in restricting the application of chemotherapy [2]. Non-operative treatment is advised when tumor involvement has not led to spinal instability, neurological involvement, or intractable pain unresponsive to medical management and includes radiotherapy, chemotherapy, hormonal therapy, and high-dose steroid therapy. Palliative radiotherapy in the form of external beam radiotherapy is an effective option for pain relief in patients with painful bone metastases. Bisphosphonates, zoledronic acid, and denosumab can also be used in metastatic spine disease to reduce the incidence of skeletal-related events like fractures and cord compressions, although it is not a primary method of treatment [11]. Surgery is generally palliative and aims to relieve the pain and restore some function [12]. The efficacy of these management modalities depends upon histological tumor type, tumor stage, and tumor spread. Indications for treatment are not controlled simply by neurological symptoms but also by quality of life. The clinical decision-making process from an oncology standpoint is further hampered, as surgical options are often inappropriate secondary to patients' comorbidities and reported to be only selectively possible in patients meeting various conditions and recommended chemotherapy or radiotherapy [13,5]. The optimal treatment for UC with bone metastasis remains divergent, and each case should be discussed individually, keeping in mind various clinicopathological factors. The management options should be determined as part of a shared decision-making process [14].

## Conclusions

Bone metastasis from UC represents a poor prognostic factor due to high morbidity and mortality secondary to skeletal-related events. It can present as a musculoskeletal complaint like low back pain or buttock pain, and it is not uncommon for previously undiagnosed cases to present to family doctors, chiropractors, or pain specialists. Hence, physicians should be particularly suspicious of a neoplastic pathology if the patient does not respond to treatment. A multidisciplinary approach is required for the management of patients with bone metastasis to improve outcomes and quality of life.
